# Nitrogen and sulfur fertilizers promote the absorption of lead and cadmium with *Salix integra* Thunb. by increasing the bioavailability of heavy metals and regulating rhizosphere microbes

**DOI:** 10.3389/fmicb.2022.945847

**Published:** 2022-08-03

**Authors:** Shaokun Wang, Xiaoyun Niu, Dongliu Di, Dazhuang Huang

**Affiliations:** ^1^College of Landscape Architecture and Tourism, Hebei Agricultural University, Baoding, China; ^2^Hebei Key Laboratory for Tree Genetic Resources and Forest Protection, Baoding, China

**Keywords:** phytoremediation, fertilization, rhizosphere, microbial metabolic activity, microbial community structure

## Abstract

Fertilization is an effective agronomic strategy to improve the efficiency of phytoextraction by *Salix integra* Thunb. However, the specific effects of the simultaneous application of nitrogen (N) and sulfur (S) fertilizers in the rhizosphere remain unclear. We investigated the bioavailability of lead (Pb) and Cadmium (Cd) along with the microbial metabolic functions and community structure in the rhizosphere soil of *S. integra* after the application of N (0, 100, and 200 kg·ha^−1^·year^−1^) and S (0, 100, and 200 kg·ha^−1^·year^−1^) fertilizers for 180 days. The simultaneous application of N and S fertilizers significantly enhanced the absorption of Pb and Cd by *S. integra*, whereas this effect was not observed for the single application of N or S fertilizer. The contents of acid-soluble Pb and Cd in the rhizosphere soil significantly increased after either single or combined fertilize applications. The microbial metabolic activity was enhanced by the N and S fertilizers, whereas the microbial diversity markedly decreased. The metabolic patterns were mainly affected by the concentration of N fertilizer. The dominant fungi and bacteria were similar under each treatment, although the relative abundances of the dominant and special species differed. Compared to the N200S100 and N200S200 treatments, the N100S100 and N100S200 treatments resulted in fewer pathogenic fungi and more rhizosphere growth-promoting bacteria, which promoted phytoremediation by *S. integra*. Redundancy analysis indicated that the pH and nitrate content were the key factors affecting the structure of the microbial community. Collectively, the results suggest interactive effects between N and S fertilizers on the rhizosphere soil, providing a potential strategy for plant-microbial remediation by *S. integra*.

## Introduction

Numerous environmental problems have emerged with the acceleration of industrialization and urbanization, the most serious of which is heavy metal pollution (Manta et al., 2002). Lead (Pb) and cadmium (Cd) are the most significant heavy metal pollutants in China (Hu et al., 2014). Many physical, chemical, and biological processes have been developed to reduce the threats posed by Pb and Cd pollution in ecosystems and humans; however, most of these approaches are costly and cause secondary pollution (Ma et al., 2016). Due to these problems, phytoremediation, a low-cost approach that does not generate secondary contamination, has emerged as a research hotspot in recent years. Plants can absorb, transfer, and stabilize various heavy metals, making them ideal candidates for the bioremediation of heavy metals in contaminated soils (Gong et al., 2018). However, the low remediation efficiency of plants has restricted their large-scale application, because of low biomass and bioavailability of heavy metals (Zhang et al., 2019; Wan et al., 2020).

The bioavailability of heavy metals is a key factor affecting phytoremediation. Compared with other fertilizers (phosphate and potash), nitrogen (N) and sulfur (S) have greater impacts on the availability of heavy metals (Hou et al., 2017). Both N and S are essential nutrients that promote plant growth (Wang et al., 2016), and these nutrients can also alter the bioavailability of heavy metals to affect the absorption of heavy metals by plants (Wang et al., 2008). The acid-soluble is the main form of heavy metals that is absorbed by plants (Moharami and Jalali, 2015). Previous studies have shown that N fertilizer can increase the accumulation of Cd in wheat (*Triticum aestivum*) and rice (*Oryza sativa*; Wang et al., 2019, 2020). Maqbool et al. (2020) reported that different forms of N fertilizers could improve the absorption efficiency of Cd by *Solanum nigrum*. The application of moderate N fertilizer improved the phytoremediation efficiency of *Sedum alfredii* in Zn/Cd-contaminated soils (Lin et al., 2020). The absorption of Cd by *Brassica juncea* and *Nicotiana tabacum* was significantly promoted by the application of S fertilizer (9.98 g·kg^−1^ and 0.51 g·kg^−1^, respectively; Dede and Ozdemir, 2016; Li et al., 2019). Thus, different types and concentrations of fertilizers have different effects on phytoremediation. However, till now, studies on the effects of N and S fertilizers on phytoremediation have focused primarily on crops and hyperaccumulators, and the interactive effects of N and S fertilizers on phytoremediation by fast-growing trees remain unclear. In addition, certain parameters such as pH and the type and content of metal available in the rhizosphere environment are considered as the most important factor in phytoextraction. In the long term, N changes the form of Cd in soil, therby affecting heavy metal absorption (Wang et al., 2020). Iqbal et al. (2012) reported that the application of S fertilizer improved the extraction efficiency of Cd by *Salix*, thereby promoting the S oxidation process of microorganisms and increasing the bioavailability of Cd in the rhizosphere soil. Rhizobacteria are directly beneficial to root patterns and provide nutrients to plants (Buée et al., 2009).

Some plant growth-promoting bacteria such as *Bacillus, Ensifer* secrete IAA and can stimulate plant growth and heavy metal accumulation (Mesa et al., 2017; Sajid et al., 2020). *Geopora*, an arbuscular mycorrhizal fungi, forms symbionts with plants to improve resistance to heavy metals (Sessitsch et al., 2013; Konkolewska et al., 2020). Soil microbiota are closely associated with soil quality and fertility (Khan et al., 2016; Mitra et al., 2018; Pramanik et al., 2018). The effects of fertilization on soil microbial communities have been widely reported, and fertilizers are known to influence the size, composition, function, and activity of the soil microbial community. The forms and concentration of N also have significant effects on the composition and diversity of the soil bacterial community (Liu et al., 2018a; Zheng et al., 2019). Fierer et al. (2012) reported that the N content has a significant effect on the composition of the bacterial community but little effect on the diversity. Fertilization with S reduced soil heterogeneity by changing the soil physical and chemical properties, thereby driving variation in the bacterial community structure (Philippot et al., 2013; Masuda et al., 2016). In general, the concentrations and forms of N and S have important effects on the composition and diversity of the microbial community (Liu et al., 2018a; Wang et al., 2018a; Zheng et al., 2019). However, few studies have addressed the simultaneous application of N and S fertilizers on soil microbial communities involved in phytoextraction. These microbes might be crucial for phytoextraction in the soil (Iqbal et al., 2012; Wang et al., 2020).

*Salix integra* Thunb. grows quickly and produces a large quantify of biomass and a deep root system. Previous studies have reported that *S. integra* had stronger absorption and tolerance to various heavy metals, for example Pb, Cu, Cd, and Zn (Yang et al., 2014, 2018; Shi et al., 2017; Cao et al., 2020). Thus, *S. integra* can be used to absorb metals. Most previous studies have focused on exposure to a single fertilizer; however, the phytoextraction of Pb and Cd by *S. integra* was enhanced by the simultaneous application of N and S fertilizers but not by the single application of N or S fertilizer. In addition, our previous research showed that the phytoremediation efficiency of *S. integra* could be affected by the rhizosphere microbes and inoculation brain (Niu et al., 2020, 2021). However, the mechanism by which simultaneous N and S fertilization affects the soil physicochemical parameters and soil microbial activity and diversity remains unclear. We hypothesized that (1) N and S fertilizers have interactive effects on soil properties, and (2) the composition and function of the rhizosphere soil microbial community are affected by N and S fertilization. A better understanding of the response of soil microbial communities to N and S in the contaminated soil is also expected to provide a theoretical basis for the phytoremediation of contaminated soil using fast-growing trees.

## Materials and methods

### Study site and plant material

Experiments were carried out at the Experimental Station of Hebei Agricultural University, Baoding City, Hebei Province (E 115°42′, N 38°81′), China. Annual rainfall at this site is approximately 532 mm, and the annual mean temperature is approximately 13.4°C. The soil is a typical meadow cinnamon soil. Contaminated soils with different Pb and Cd concentrations were added artificially in 2017. The soil properties and metal concentrations were as follows: pH = 8.42, soil organic matter = 18.13 g·kg^−1^, total nitrogen = 319.9 mg·kg^−1^, total phosphorus = 205.9 mg·kg^−1^, total potassium = 6.37 mg·kg^−1^, Pb concentration = 296.4 mg·kg^−1^, and Cd concentration = 23.7 mg·kg^−1^. 1-year-old *S. integra* cuttings with similar growth and vigor were used in this study.

### Experimental setup

Experiments were carried out using pots (height = 30 cm, diameter = 30 cm) in April 2019. Every pot was filled with 20 kg of air-dried loam soil. The soil water content was maintained at 60% of its water holding capacity during the experiment. Three N and S concentrations were established according to the fertilization concentration of 1-year-old seedlings (Cheng, 2012). N was added as CO(NH_2_)_2_, and S was added as Na_2_SO_4_. The contents of applied N and S fertilizers (in terms of N and S contents) are shown in Supplementary Table S1. The experiments included nine treatments with 12 replicates per treatment.

Fertilizer was added every 15 days from May 15 to July 15. For the N100 and N200 fertilization levels, 0.3031 and 0.6062 g CO(NH_2_)_2_, corresponding to 0.1413 and 0.2826 g N, respectively, were applied each time. For the S100 and S200 fertilization levels, 0.6270 and 1.2540 g Na_2_SO_4_, corresponding to 0.1413 and 0.2826 g S, respectively, were applied. The fertilizers were dissolved in water and then sprayed on the soil surface of each pot. All pots were placed randomly and subjected to the same treatment conditions (watering, soil loosening, and weeding) during the growing season.

### Plant and soil sampling

After growth for 180 days, the rhizosphere and bulk soils of the plants (three replicates each) were collected for each treatment in October 2019. The roots, stems, and leaves were harvested separately. The roots were soaked in 25 mmol·L^−1^ Na_2_-EDTA solution for 15 min to remove heavy metal ions adsorbed on the surface and then gently washed with distilled water. To sample the rhizosphere soil, the plants were removed from the pots and shaken to remove large soil aggregates. The rhizosphere soil was obtained using a brush that attached firmly to the root surface. Five randomly selected roots were pooled into a single sample, and approximately 3 g of soil for molecular analyses was stored at −80°C until DNA extraction. Residual soils for measuring the soil chemical properties and the contents of Pb and Cd were air-dried. All tools were sterilized between the sampling of different rhizosphere soils.

### Soil chemical properties

The soil was air-dried and passed through a 60-mesh sieve. After digestion with concentrated sulfuric acid, the organic matter content (SOM) was determined by the potassium dichromate heating method. Available phosphorus (AP) was determined by the molybdenum antimony colorimetric method after extraction with sodium bicarbonate. Available potassium (AK) was determined with an atomic spectrophotometer after extraction with acetic acid. After dissolving the soil in deionized water, the pH (1:2.5 m/V) was measured using an electronic pH meter, nitrate N was determined using phenol disulfonic acid colorimetry, ammonium N was determined using KCl leaching-indophenol blue colorimetric method, available sulfur (AS) was determined using the phosphate extraction-barium sulfate turbidimetric method, and cation exchange capacity (CEC) was determined using the chlorine barium-sulfuric acid exchange method (Lu, 2000).

### Determination of heavy metals in plant and soil samples

Dried and homogenized *S. integra* tissue powders were thoroughly digested with HNO_3_ and HClO_4_ (10:1, v/v). The samples were then analyzed using an atomic absorption spectrometer (Shimadzu AA-680, Kyoto, Japan).

The soil (0.5 g) was digested in 10 ml of a mixture of HNO_3_ and HClO_4_ (10:1, v/v) *via* microwave digestion (Sined MDS-6, Shanghai, China) at 180°C until the solution become transparent (approximately 3.5 h). The solution was then filtered through a 0.45-μm membrane and diluted to 50 ml. Soil samples were analyzed by atomic absorption spectrophotometry.

Soil Pb and Cd fractions were collected by BCR sequential extraction using a soil sample (0.5 g) containing the acid-soluble, reducible, oxidizable, and residual fractions (Rauret et al., 1999). The acid-soluble fraction was extracted with 0.11 mol·L^−1^ acetic acid for 16 h. The reducible fraction was extracted with 0.5 mol·L^−1^ hydroxylamine hydrochloride for 16 h (pH = 1.5). The oxidizable fraction was extracted using 8.8 mol·L^−1^ H_2_O_2_ at 85°C for 2 h. The residual fraction was obtained by extraction with 1.0 mol·L^−1^ ammonium acetate and digested by adding three acids (2 mlHNO_3_, 4 ml HCl, and 2 mL HF). The different fractions were evaluated by atomic absorption spectrophotometry.

### Community-level physiological profiling

Community-level physiological profiling (CLPP) was used to assess the metabolic function and functional diversity of the microbial community (Ma et al., 2018; Zhao et al., 2018). The carbon source utilization pattern (CSUP) of the soil microbial community was assessed using a Biolog ECO microplate (Category No. 1506, United States). Detailed information about the experimental procedure can be found in Niu et al. (2019). For further analysis of the CLPP data, the absorbance reading at 240 h was identified as the time point for all samples. The data were analyzed to determine metabolic diversity indices according to Sofo et al. (2018).

### Microbial community analysis

The structures of the rhizosphere bacterial and fungal communities were analyzed for five treatments: the control (N0S0), two treatments that enhanced the absorption of heavy metals by plants (N100S100 and N100S200), and two treatments that suppressed the absorption of heavy metals by plants (N200S100, and N200S200). Three replicates for each of the five treatments were analyzed, resulting in a total of 15 samples analyzed. Extracted total DNA was prepared for the analysis of microbial community structure *via* high-throughput sequencing. Microbial DNA was extracted from each fresh soil sample using a Fast Soil DNA kit (Omega Biotek, Inc.) following the manufacturer’s protocol. DNA samples were quantified using a nano spectrophotometer (Thermo Scientific, Wilmington, DE, United States) and 1% (w/v) agarose gel to verify the concentration and purity. The V3–V4 hypervariable region of the bacterial 16S rRNA gene was selected for PCR amplification with the primers 341F (5-CCTAYGGGRBGCASCAG-3′) and 806R (5′-GGAC TACNNG GGTAT-CTAAT-3′) for bacterial identification. The internal transcribed spacer (ITS) hypervariable regions of the fungal genes were amplified with primers ITS5-1737F (GGAAGTAAAAGTCGTAACAAGG) and ITS2-2043R (GCTGCGTTCTTCA TCGTGC) for fungal identification. Details of the PCR program are described in Xu et al. (2018). Each sample was amplified three times. The amplified products were mixed in equal amounts and then sequenced using the Novaseq 6,000 PE 250 platform (Illumina, United States; Allwegene Biotech Co., Ltd., Tianjin, China). Raw sequences of 16S rRNA and ITS gene have been submitted to NCBI Sequence Read Archive (Nos. PRJNA846123 and PRJNA846147, respectively).

The raw fastq files were demultiplexed and quality filtered using QIIME version 1.9.1 according to Caporaso et al. (2011). In subsequent analyses, reads were truncated at any site with an average quality score < 20 over a 50-bp sliding window and without ambiguous bases. Sequences with overlap longer than 10 bp were merged by overlapping sequences. UPARSE software (version 7.1) was used to assemble the operational taxonomic units (OTUs) at 3% evolutionary sites, and UCHIME software was used to identify and remove chimeric sequences. The taxonomic identity of each 16S rRNA and each ITS gene sequence was determined using the Ribosomal Database Project classifier with 70% confidence intervals for the Silva (Release 128) 16S rRNA and Unite (Release 7.0) databases, respectively. The OTUs were then analyzed. Alpha diversity was calculated using Mothur (version 1.30.1), while the beta diversity metrics were calculated using QIIME and the R base package (Oksanen et al., 2013). We also estimated alpha diversity using the Chao1 and Shannon indexes. The co-occurrence networks were constructed in this study. Networks were constructed based on Spearman correlation (*r* > 0.7 and *p* < 0.05) using the R package igraph (1.2.5). The division of network modules was based on the greedy optimization of the modularity algorithm. All networks were visualized using Gephi software (0.9.2).

### Statistical analysis

Each data point represents the average value ± standard error for three replicates. The effects of different N and S fertilizer treatments were evaluated by two-way analysis of variance (ANOVA). The Shapiro–Wilk test was used to test the normality of the analytical features. According to the distributions of the estimated parameters, the least significant difference method was used for parametric tests, while the Kruskal–Wallis method was used for nonparametric tests. SPSS 19.0 (IBM, America) was used for all statistical analyses, with *p* < 0.05 indicating significant difference. Redundancy analysis (RDA) was performed using CANOCO software (Windows version 4.5) to determine the relationship between soil properties and microbial communities. The differences in community composition were visualized by the non-metric multidimensional scaling (NMDS) method using the vegan package meta-MDS function in R. Variance partial analysis (VPA) with RDA (X, Y, Z) was carried out in the vegan package to analyze the effects of main environmental factors and co-environmental factors on species distribution and determine the contributions of various environmental factors to species distribution. Structural equation modeling (SEM) performed using the robust maximum likelihood estimation in AMOS 24.0 (Amos, Development Corporation, Meadville, PA, United States).

## Results

### Total absorption of Pb and Cd

**Figure 1:**
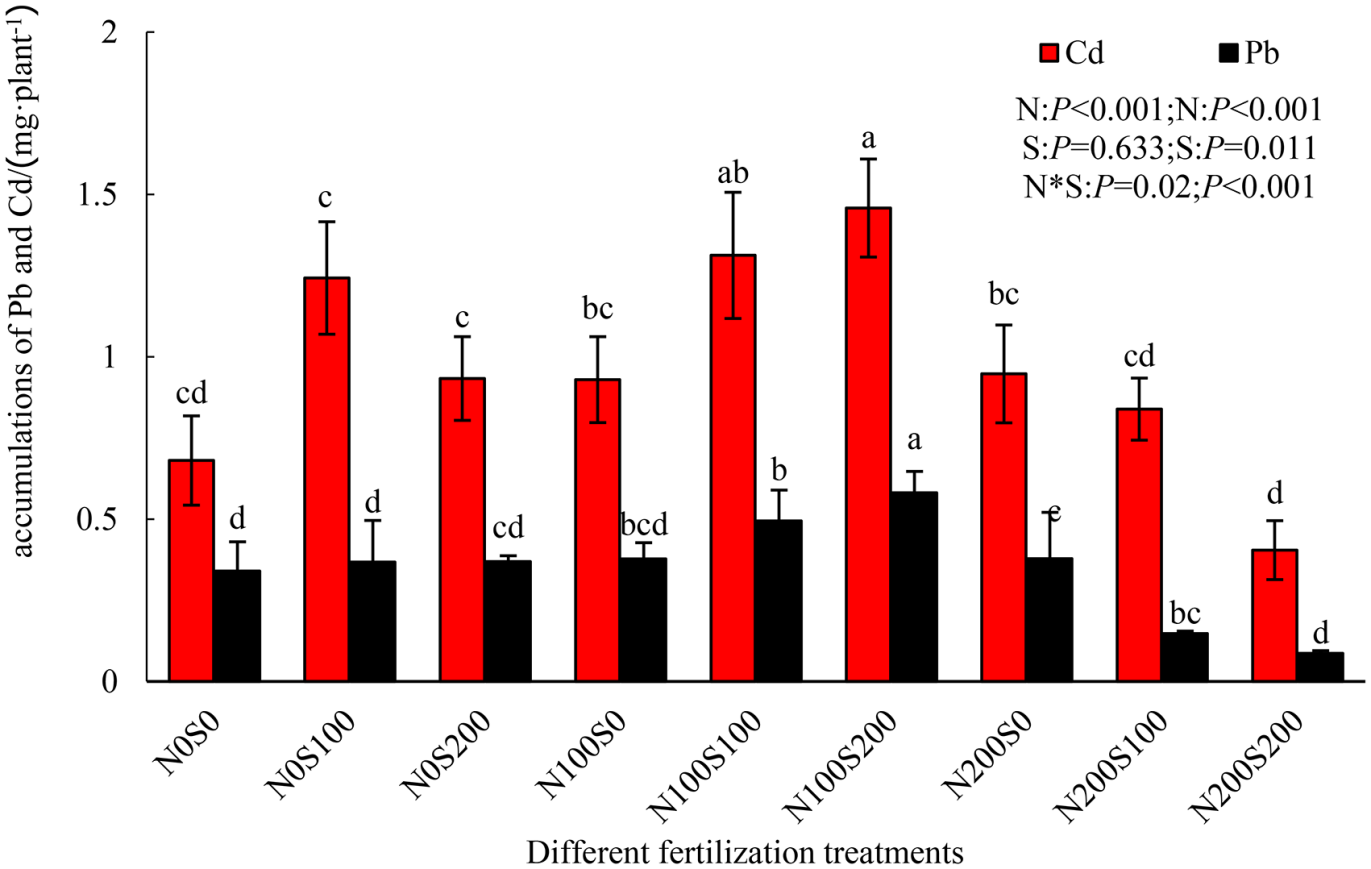
Effect of different fertilization treatments on the total accumulation of Pb and Cd of *Salix integra* Thunb. Bars with different letters represent statistically significant differences.

The simultaneous application of N and S fertilizers significantly enhanced the absorption of Pb and Cd by *S. integra*, whereas the application of N or S fertilizer alone did not have this effect (Figure 1). The N and S fertilizers had an interactive effect on the absorption of Pb and Cd. Among the treatments, the total absorptions of Pb (0.58 mg·plant^−1^) and Cd (1.46 mg·plant^−1^) by *S. integra* were highest under the N100S200 treatment (71.11 and 114.17% compared with the control, respectively). In contrast, the total absorptions of Pb (0.08 mg·plant^−1^) and Cd (0.40 mg·plant^−1^) were lowest under the N200S200 treatment (not significantly different compared to the control).

### Soil properties

The single or simultaneous application of N and S fertilizers significantly reduced the pH (Table 1), with the lowest pH (7.84) observed under the N200S100 treatment. Interactive effects between N and S fertilization were observed on pH, CEC, and the AP, AK, nitrate, and AS contents. The single application of N fertilizer and the simultaneous application of N and S fertilizers significantly reduced the content of SOM, with the lowest value (10.11 g·kg^−1^) observed under the N200S100 treatment. The simultaneous application of N and S fertilizers significantly increased the CEC and AP content, with the largest values (13.24 cmol·kg^−1^ and 43.36 mg·kg^−1^, respectively) observed under the N200S200 treatment. However, the simultaneous application of N and S fertilizers significantly reduced the content of AK, with lowest value (110.95 mg·kg^−1^) observed under the N200S200 treatment. The application of N and S fertilizers significantly increased the content of nitrate, which was highest (105.24 mg·kg^−1^) under the N200S200 treatment. However, the single application of N fertilizer or the simultaneous application of N and S fertilizers significantly reduced the content of ammonium, which was lowest (23.71 mg·kg^−1^) under the N200S0 treatment. The application of N and S fertilizers significantly increased the content of AS, which was highest (40.81 mg·kg^−1^) under the N200S0 treatment.

**Table 1:** Effect of different fertilization treatments on rhizosphere soil chemical properties.

Treatmens	pH	SOM /g·kg^−1^	CEC /cmol·kg^−1^	AP /mg·kg^−1^	AK /mg·kg^−1^	Nitrate /mg·kg^−1^	Animonium /mg·kg^−1^	AS /mg·kg^−1^
CK	8.12 ± 0.05a	14.31 ± 0.59a	11.14 ± 0.34bc	19.45 ± 1.71b	134.79 ± 7.19ab	16.13 ± 4.33d	25.75 ± 0.36a	10.89 ± 1.57d
N0S100	7.9 ± 0.11c	12.7 ± 0.93a	10.78 ± 0.44bc	18.42 ± 1.39b	130.89 ± 1.75b	36.03 ± 13.77bc	28.29 ± 2.56a	37.41 ± 8.67ab
N0S200	7.91 ± 0.02bc	12.99 ± 1.08a	10.45 ± 0.45c	23.79 ± 1.81b	145.26 ± 2.81a	22.57 ± 0.24d	27.59 ± 0.4a	21.53 ± 0.23 cd
N100S0	7.92 ± 0.03bc	14.1 ± 1.42a	11.5 ± 0.4b	21.48 ± 0.98b	139.12 ± 0.54ab	42.95 ± 3.55b	26.9 ± 0.68a	22.06 ± 1.97 cd
N100S100	8.05 ± 0.01a	12.8 ± 1.42a	10.6 ± 0.12bc	22.55 ± 0.9b	135.25 ± 2.34ab	37.39 ± 0.8bc	25.83 ± 1.06a	38.09 ± 14.22ab
N100S200	8.09 ± 0ab	12.19 ± 0.6a	10.19 ± 0.78c	19.73 ± 1.27b	109.56 ± 11.72c	37.01 ± 1.54bc	25.41 ± 0.32a	28.19 ± 0.76abc
N200S0	7.89 ± 0.01c	11.33 ± 0.84b	10.84 ± 0.35bc	20.83 ± 0.8b	110.15 ± 0.89c	25.38 ± 8.3 cd	23.71 ± 0.18b	40.81 ± 0.21a
N200S100	7.84 ± 0.01c	10.11 ± 0.59b	11.09 ± 0.05bc	21.15 ± 0.93b	114.7 ± 0.8c	95.33 ± 2.29a	24.26 ± 1.43b	17.13 ± 0.76 cd
N200S200	7.86 ± 0.03c	10.35 ± 0.34b	13.24 ± 0.36a	43.36 ± 6.73a	110.95 ± 2.46c	105.24 ± 0.34a	25.61 ± 0.99b	27.57 ± 2.63bc
*P*(N)	*	*	*	*	*	*	*	ns
*P*(S)	*	*	ns	*	ns	*	ns	ns
*P*(N*S)	*	ns	*	*	*	*	ns	*

*N* and *S* represent *N* and *S* main effect respectively, N*S represents the interactive effect, Different letters indicate that the values differ significantly at *p* < 0.05. *indicates *p* < 0.05, ns indicates no significant difference.

### Partitioning of Pb and Cd in soil

**Figure 2:**
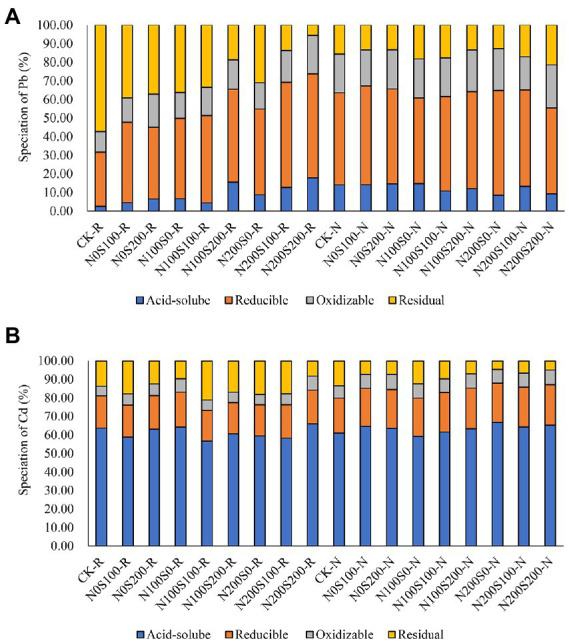
The content of different speciation Pb **(A)** and Cd **(B)** in rhizosphere and bulk soil under different treatments. CK rhizosphere soil (C-R), CK bulk soil (C-N), the same below.

The relative contents of the four different forms of Pb and Cd were similar under different treatments, but the main forms of the metals were different (Figure 2). The contents of reducible Pb and Cd were relatively high, whereas the contents of acid-soluble, oxidizable, and residual Pb and Cd were relatively low. The application of N and S fertilizers increased the proportion of acid-soluble Pb in rhizosphere soil and reduced the proportion of residual Pb, but this effect was not obvious in bulk soil. Apart from the N100S0 treatment, the application of N and S fertilizers increased the relative content of acid-soluble Cd in bulk soil.

### Microbial metabolism

#### Metabolic activity of the microbial community

**Figure 3:**
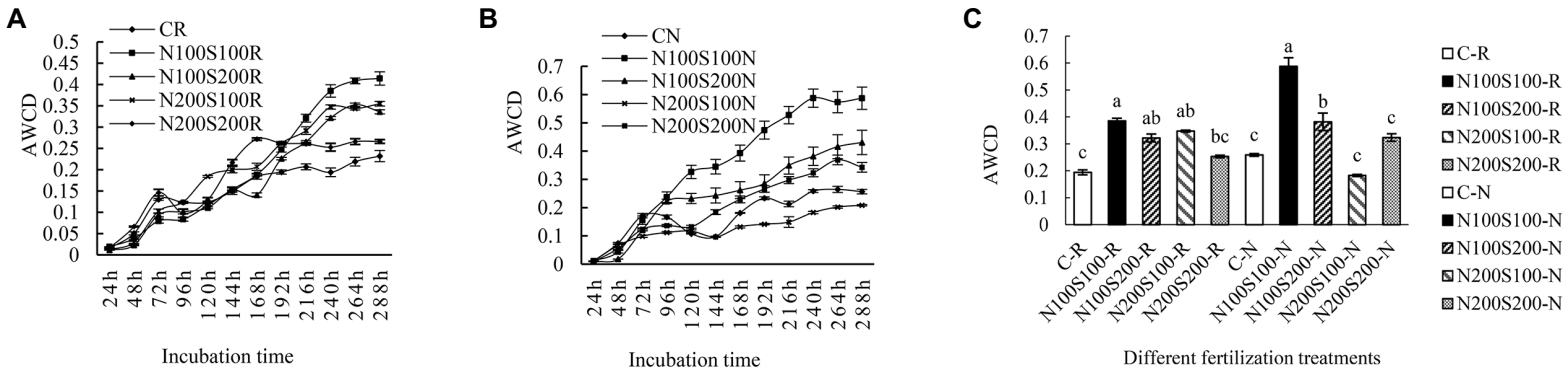
Effect of different fertilization treatments on microbial community metabolic activity. AWCD changes of microbial community in rhizosphere **(A)**, AWCD changes of microbial community in bulk soil **(B)**, microbial 240 h AWCD value **(C)**, CK rhizosphere soil (C-R), CK bulk soil (C-N), the same below. Different letters indicate that the values differ significantly at *p* < 0.05.

Microbial metabolic activity is reflected by average well color development (AWCD). The change in AWCD during the initial 24 h was relatively small; subsequently, AWCD gradually increased with incubation time (Figure 3). The trend of AWCD value was similar among the different treatments. The application of N and S fertilizers significantly enhanced the metabolic activity of the microbial community and increased the AWCD in the bulk soil. The rate of carbon source utilization was basically stable at 240 h; thus, the AWCD values at 240 h were used for further analysis. In the rhizosphere and bulk soils, the AWCD values were significantly higher under the N100S100 and N100S200 treatments compared to the control. The AWCD values under the other treatments did not differ significantly from that of the control.

#### Utilization of specific carbon sources

**Figure 4:**
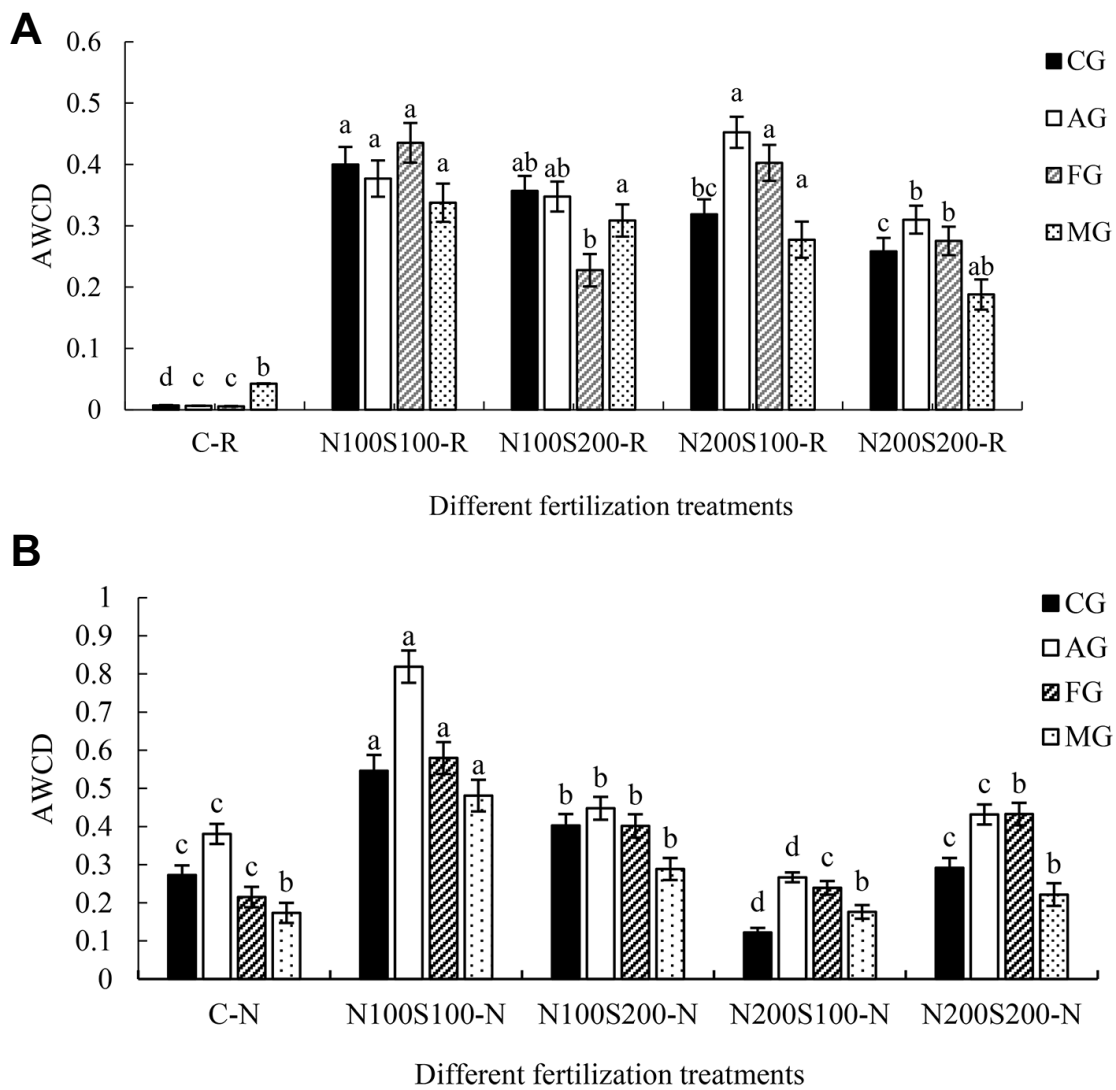
Utilization of the carbon source groups of microbial community. Utilization of the carbon source groups of microbial community in rhizosphere **(A)**, utilization of the carbon source groups of microbial community in bulk soil **(B)**, carbohydrates and their derivatives (CG), amino acids and their derivatives (AG), fatty acids and lipids (FG), metabolic regulation and secondary metabolites (MG). Different letters indicate that the values differ significantly at *p* < 0.05.

We classified the 31 carbon sources into four categories based on the microbial metabolic pathways of the three major nutrients: carbohydrates and their derivatives (CG, 12 kinds); amino acids and their derivatives (AG, 6 kinds); fatty acids and lipids (FG, 5 kinds); and metabolic regulation and secondary metabolites (MG, 8 kinds; Xun et al., 2018). The utilization rates of the specific carbon sources varied among the different treatments. In rhizosphere soil, the simultaneous application of N and S fertilizers significantly increased the utilization of CG, AG, FG, and MG compared with the control (Figure 4A). In bulk soil, the N100S100 and N100S200 treatments increased the utilization rates of CG, AG, and FG compared to the control (Figure 4B), while the N200S100 treatment significantly decreased the utilization of CG and AG, and the N200S200 treatment significantly increased the utilization of FG.

#### Metabolic profile of the microbial community

**Figure 5:**
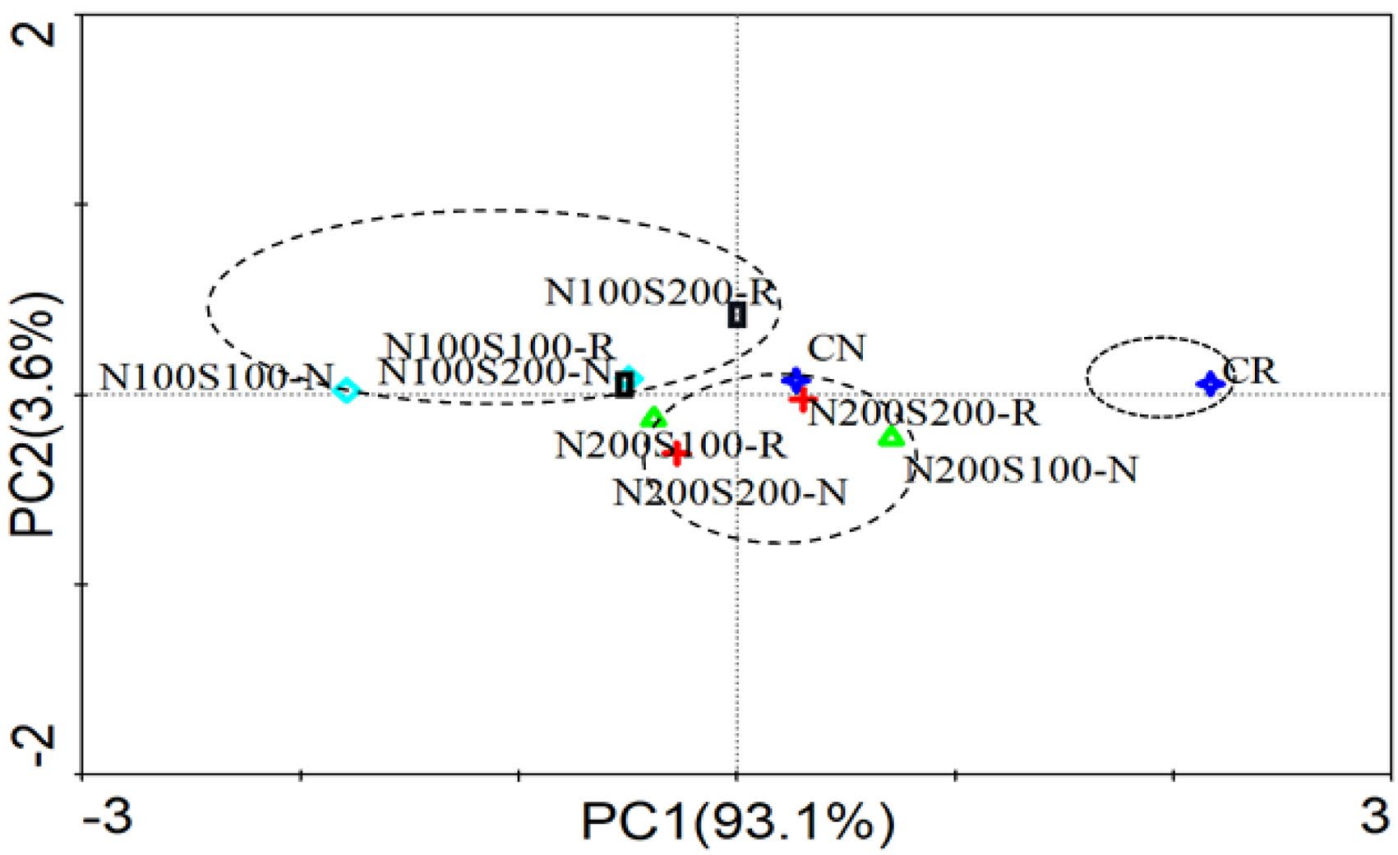
Principal component analysis basing carbon source utilization patterns of microbial community.

Principal component analysis was used to visualize the CSUPs of the microbial communities (Figure 5). The contribution of the first principal component (PC1) to the variance in the data was 93.1%, while the second principal component (PC2) accounted for 3.6% of the variance. All samples were distributed along the positive direction of the PC1 axis. The carbon sources utilized by the microorganisms under different treatments could be divided into three groups according to the N concentration. The first group contained the CK rhizosphere soil. The bulk CK soil and N200S100 N200S200 treatments were close to the second group, which included the rhizosphere and bulk soils. The third group contained the rhizosphere and bulk soils under the N100S100 and N100S200 treatments. The microbial metabolism also showed significant differences between the rhizosphere and bulk soils for the same treatment. Fertilization and root activity have positive effects on microbial metabolism.

### Microbial community

#### Richness and diversity of the microbial community

The results indicated a good coverage of the bacterial and fungal communities (between 0.98 and 0.998), suggesting that most of the fungal and bacterial taxa were detected in all soil samples (Tables 2, 3). Compared with the control, the diversity (characterized by the Shannon index) and richness (characterized by the Chao 1 index) of the fungal and bacterial communities were significantly reduced by the simultaneous application of N and S fertilizers. Among all treatments, the fungal community diversity and richness were lowest under the N100S100 treatment (Shannon index = 2.66, Chao1 index = 479.09), while the bacterial community diversity and richness were lowest under the N100S200 treatment (Shannon index = 9.40, Chao1 index = 3831.89).

**Table 2:** Effect of different fertilization treatments on alpha diversity indices of fungi community.

Index	Treatments					*P*
	N0S0	N100S100	N100S200	N200S100	N200S200	
Observed_species	842 ± 87.54a	424.33 ± 54.78c	487.33 ± 186.75bc	536 ± 48.28bc	643.33 ± 79.14b	*
Shannon	4.83 ± 0.53a	2.66 ± 0.32b	2.74 ± 1.22b	3.08 ± 0.46b	4.17 ± 1.05ab	*
Simpson	0.86 ± 0.05	0.645 ± 0.09	0.61 ± 0.20	0.70 ± 0.13	0.81 ± 0.15	ns
Chao1	911.12 ± 94.82a	479.09 ± 47.82a	538.22 ± 189.35c	611.77 ± 28.86ab	716.88 ± 84.19ab	*
ACE	923.12 ± 96.63a	495.27 ± 49.09a	555.49 ± 195.49b	614.96 ± 26.60ab	724.38 ± 83.74ab	ns
Good coverage	0.99 ± 0.001	0.998 ± 0	0.99 ± 0.001	1.00 ± 0	0.998 ± 0	ns
PD whole tree	344.7 ± 24.45	171.02 ± 19.09	226.07 ± 82.83	213.91 ± 6.43	274.92 ± 49.79	ns

Different letters indicate that the values differ significantly at *p* < 0.05. *indicates *p* < 0.05, ns indicates no significant difference.

**Table 3:** Effect of different fertilization treatments on alpha diversity indices of bacteria community.

Index	Treatments					*P*
	N0S0	N100S100	N100S200	N200S100	N200S200	
Observed_species	3,827.33 ± 160.58a	3,636.33 ± 100.32ab	3,425.33 ± 162.46bc	3,577 ± 16.64c	3,799.33 ± 121.54ab	*
Shannon	9.88 ± 0.11a	9.62 ± 0.16ab	9.40 ± 0.31c	9.70 ± 0.09ab	9.93 ± 0.09a	*
Simpson	0.99 ± 0.00	1.00 ± 0.001	0.99 ± 0.00	1.00 ± 0.00	1.00 ± 0	ns
Chao1	4,349.90 ± 249.18a	4,396.74 ± 478.45a	3,831.89 ± 145.24c	4,059.99 ± 30.33ab	4,274.80 ± 150.75ab	*
ACE	4,455.48 ± 252.98	4,485.38 ± 478.35	3,900.92 ± 140.07	4,156.51 ± 58.42	4,352.16 ± 152.00	ns
Good coverage	0.98 ± 0.00	0.98 ± 0.00	0.99 ± 0.00	0.99 ± 0.00	0.9 ± 0.00	ns
PD whole tree	252.17 ± 8.66	241.65 ± 11.26	233.42 ± 27.43	240.30 ± 4.93	250.21 ± 10.15	ns

Different letters indicate that the values differ significantly at *p* < 0.05. *indicates *p* < 0.05, ns indicates no significant difference.

#### Composition and co-occurrence network of bacterial and fungal communities

**Figure 6:**
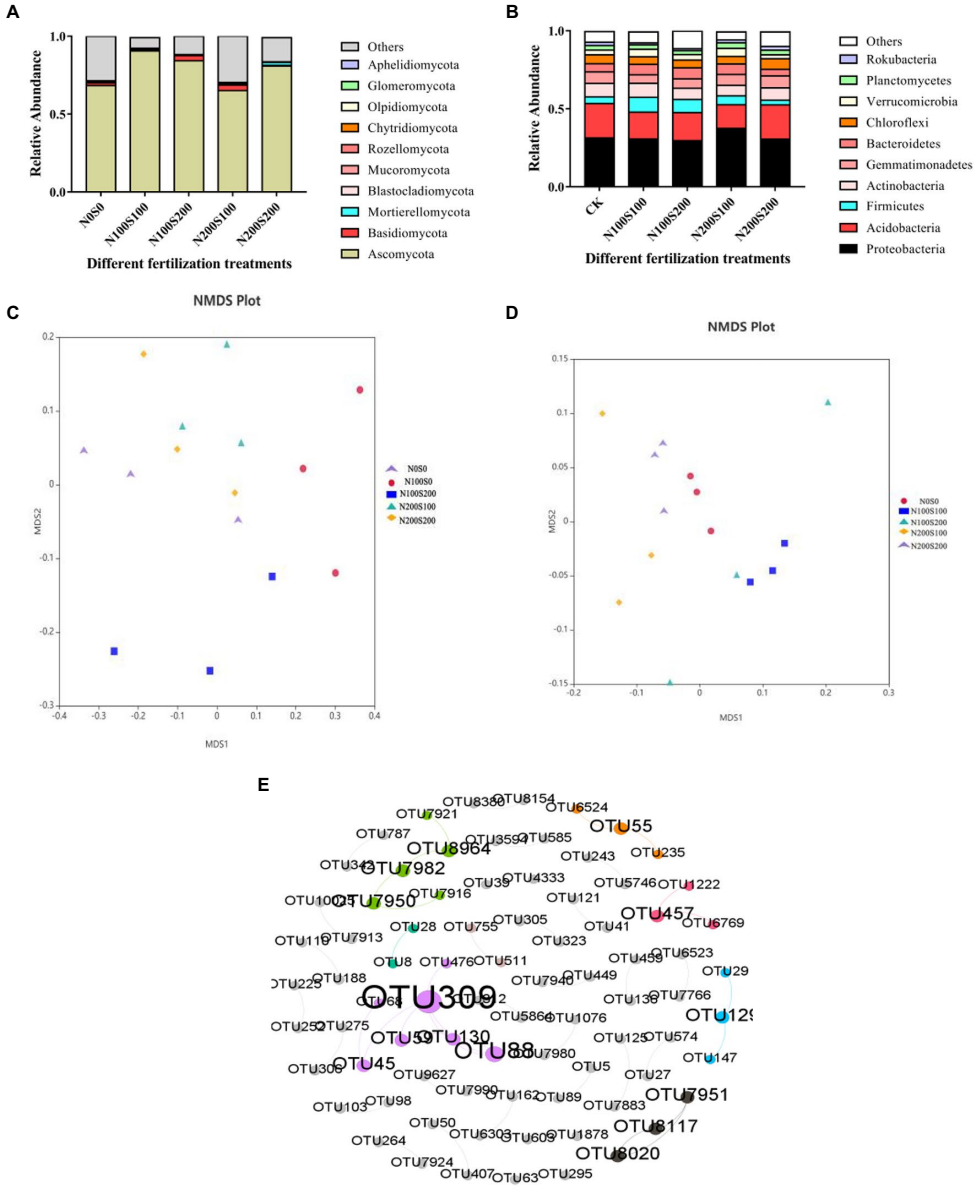
Effect of different fertilization treatments on fungi and bacterial community structure. The relative abundance of fungi and bacteria in rhizosphere soil with different treatments at phylum level [fungi **(A)**, bacteria **(B)**]; NMDS ordination plots derived from Unifrac distance matrix [fungi **(C)**, bacteria **(D)**], Co-Occurrence Patterns of bacteria and fungi **(E)**.

The fungi belonged to 13 phyla (Figure 6A). While the dominant phyla were similar among the different treatments, their relative abundances differed (Table 4). Among the phyla, the relative abundance of *Ascomycota* was the highest, exceeding 65% in each treatment. The applied N and S fertilizers had significant interactive effects on the relative abundances of *Ascomycota* and *Mucoromycota*; their application significantly increased the relative abundance of *Ascomycota* and significantly decreased the relative abundance of *Mucoromycota*. Compared with the control, the relative abundance of *Mortierellomycota* increased significantly under N200S200 treatment. The bacteria belonged to 30 phyla (Figure 6B). As for the fungi, the dominant phyla (relative abundance >1%) were similar under the different treatments, while their relative abundances differed (Table 5). Among all phyla, *Proteobacteria* had the highest abundance in all samples followed by *Acidobacteria*. The applied N and S fertilizers had significant interactive effects on the abundances of *Proteobacteria*, *Acidobacteria*, *Actinobacteria*, *Bacteroidetes* and *Chloroflexi*; the simultaneous application of N and S fertilizers significantly increased the relative abundances of *Proteobacteria*, *Firmicutes*, *Bacteroidetes* and *Verrucomicrobia* but suppressed the relative abundances of *Acidobacteria*, *Actinobacteria*, *Gemmatimonadetes*, *Chloroflexi* and *Rokubacteria*. Furthermore, the relative abundances of bacteria were affected by the concentrations of N and S. For example, the relative abundances of *Firmicutes*, *Gemmatimonadetes*, *Chloroflexi*, and *Rokubacteria* were significantly reduced under N100S100 treatment compared the control, whereas no significant differences were observed under N200S200 treatment. Compared with the control, the relative abundance of *Verrucomicrobia* was significantly increased under N100S100 treatment, but the difference was not significant under N200S200 treatment.

**Table 4:** The relative abundance of main fungi phyla in different samples.

Phyla	*Ascomycota*		*Basidiomycota*	*Mortierellomycota*	*Blastodadiomycota*	*Mucoromycota*	*Rozellomycota*	*Chytridiomycota*
N0S0	0.6833 ± 0.0512b		0.0164 ± 0.0007	0.0051 ± 0.0006b	0.0003 ± 0	0.0068 ± 0.0004a	0.0006 ± 0.0001	0.0013 ± 0.0003
N100S100	0.9056 ± 0.0216a		0.0083 ± 0.0008	0.0073 ± 0.0058b	0.0004 ± 0.0005	0.0001 ± 0.0001c	0.0004 ± 0.0003	0.0003 ± 0.0001
N100S200	0.8422 ± 0.0942a		0.0331 ± 0.0301	0.0051 ± 0.0037b	0.0006 ± 0.0006	0 ± 0.0001c	0.0005 ± 0.0004	0.0004 ± 0.0001
N200S100	0.6511 ± 0.0347a		0.035 ± 0.0388	0.0079 ± 0.0045b	0.0014 ± 0.0011	0.0041 ± 0.0002b	0.0012 ± 0.0005	0.0019 ± 0.0018
N200S200	0.8079 ± 0.0168a		0.0054 ± 0.0016	0.018 ± 0.0024a	0.0008 ± 0.0005	0.0008 ± 0.001c	0.0009 ± 0.0003	0.0011 ± 0.0012
*P*(N)	0.003		0.976	0.032	0.217	<0.001	0.049	0.127
*P*(S)	0.232		0.88	0.175	0.722	0.001	0.742	0.657
*P*(N*S)	0.013		0.11	0.044	0.33	0.001	0.399	0.501

Different letters indicate that the values differ significantly at *p* < 0.05.

**Table 5:** The relative abundance of main bacteria phyla in different samples.

Phyla	*Proteobacteria*	*Acidobacteria*	*Firmicutes*	*Actinobacteria*	*Gemmati monadetes*	*Bacteroidetes*	*Chloroflexi*	*Verruco microbia*	*Plancto mycetes*	*Roku bacteria*
CK	0.31311 ± 0.00042b	0.2196 ± 0.00085a	0.0433 ± 0.00577bc	0.08608 ± 0.00395ab	0.07392 ± 0.00796a	0.05273 ± 0.01022bc	0.05651 ± 0.00343a	0.0309 ± 0.00131b	0.02965 ± 0.00217	0.02006 ± 0.00196a
N100S100	0.30666 ± 0.00419b	0.1716 ± 0.00556bc	0.09432 ± 0.00324a	0.09 ± 0.00555a	0.05542 ± 0.00369b	0.0666 ± 0.009ab	0.04848 ± 0.00122b	0.04805 ± 0.00867a	0.02804 ± 0.00055	0.0127 ± 0.00086b
N100S200	0.29559 ± 0.00747b	0.18017 ± 0.00178b	0.08413 ± 0.01422a	0.07121 ± 0.00138bc	0.05977 ± 0.00846b	0.07119 ± 0.00601a	0.05027 ± 0.00107b	0.03465 ± 0.00411a	0.02474 ± 0.0012	0.01291 ± 0.00099b
N200S100	0.37395 ± 0.01875a	0.15137 ± 0.02095c	0.05742 ± 0.00665b	0.06646 ± 0.01228c	0.07013 ± 0.01082a	0.06768 ± 0.00462ab	0.04804 ± 0.0003a	0.05202 ± 0.00384b	0.03594 ± 0.00699	0.0183 ± 0.00157a
N200S200	0.30563 ± 0.00068b	0.21858 ± 0.0063a	0.03059 ± 0.00513c	0.07859 ± 0.00274abc	0.07679 ± 0.00483a	0.04249 ± 0.00409c	0.06778 ± 0.0051a	0.02537 ± 0.00143b	0.02989 ± 0.00522	0.02352 ± 0.00375a
*P*(N)	<0.001	0.233	<0.001	0.106	0.015	0.022	0.002	0.444	0.047	<0.001
*P*(S)	<0.001	<0.001	0.008	0.481	0.33	0.071	<0.001	<0.001	0.134	0.098
*P*(N*S)	0.001	0.002	0.17	0.007	0.835	0.015	0.01	0.074	0.642	0.123

Different letters indicate that the values differ significantly at *p* < 0.05.

NMDS analysis (stress = 0.113; stress = 0.102) revealed obvious differences in microbial community structure among the treatments (Figures 6C,D). The microorganisms formed different clusters under the different fertilization treatments, indicating that N and S fertilizers had interactive effects on the fungal and bacterial community structures. The fungal communities formed similar clusters under the N100S100 and N100S200 treatments, indicating that the N fertilizer had a stronger effect on the fungal community than the S fertilizer.

Based on an examination of the co-occurrence patterns of OTUs in the meta-network of bacteria and fungi (Figure 6E), we found a certain correlation between bacteria and fungi (Supplementary Table S2), The degree of connection was low, which may be due to the increased activity of heavy metals after fertilization, thereby weakening the connection between microorganisms. We identified two keystone taxa (nodes with a high value of both betweenness centrality and closeness centrality were defined as keystone taxa):TUO309 (g__Marinobacter) and OTU45(g__Bacillus).

#### Specific microbial species under different treatments

At the genus level, metastasis was used to analyze special microbial species with significant changes in relative abundance between treatments. Two-way ANOVA was used to analyze the effects of N and S fertilizers on these special species with the relative abundance of the bacteria and top 12 fungi in each sample. A total of 13 fungi genera were included (Table 6). The abundances of *Gaeumannomyces*, *Pseudallescheria*, *Russula* and *Fusarium* were lower under the N100S100 and N100S200 treatments than under the N200S100 and N200S200 treatments. The simultaneous application of N and S fertilizers reduced the relative abundances of *Geopora*, *Dactylonectria*, *Sirastachys*, *Neocosmospora*, *Beauveria*, *Aspergillus* and *Aureobasidium*, which were lower under the N100S100 and N100S200 treatments compared to under the N200S100 and N200S200 treatments. A total of 16 genera of bacteria were included. As shown in Table 7, the simultaneous application of N and S fertilizers increased the relative abundances of *Ensifer*, *Iamia*, *Lachnoclostridium*, *Lacibacter*, *Niastella*, *Streptomyces* and *unidentified_Nitrospiraceae*, which were more enriched in the promotive treatments (N100S100 and N100S200) compared with the suppressive treatment. The simultaneous application of N and S fertilizers reduced the relative abundances of *Acidibacter*, *Faecalibaculum*, *Gemmatimonas* and *Haliangium*, which were greater under the N100S100 and N100S200 treatments than under the N200S100 and N200S200 treatments.

**Table 6:** Effect of different fertilization treatments on the relative abundance of specific fungi assemblages.

		*Geopora*	*Dactylo nectria*	*Gaeu mannomyces*	*Histoplasma*	*Moeszio myces*	*Pseudalle scheria*	*Sirastachys*	*Russula*	*Neocos mospora*	*Beauveria*	*Aspergillus*	*Fusarium*	*Aureoba sidium*
Treatments	CK	0.3747 ± 0.10085a	0.00497 ± 0.00188a	0.00025 ± 0.00003b	0.00007 ± 0.00001ab	0.00001 ± 0.00001ab	0.00008 ± 0.00003b	0.00554 ± 0.00258a	0 ± 0c	0.02369 ± 0.01602a	0.00018 ± 0.00005a	0.01666 ± 0.00151a	0.01296 ± 0.00164bc	0.00027 ± 0.00009a
	N100S100	0.25156 ± 0.03142b	0.00113 ± 0.00011b	0.00017 ± 0.00003b	0.00001 ± 0.00001b	0.00001 ± 0.00001b	0.00003 ± 0.00001b	0.00046 ± 0.00015b	0.00001 ± 0.00001bc	0.01236 ± 0.00297b	0 ± 0b	0.00006 ± 0.00001c	0.00403 ± 0.00008c	0 ± 0b
	N100S200	0.16716 ± 0.03514b	0.00147 ± 0.00047b	0.00007 ± 0.00004b	0.00006 ± 0.00004ab	0.00002 ± 0.00001b	0 ± 0b	0.00019 ± 0.00002b	0.00001 ± 0.00001bc	0.01423 ± 0.0063b	0.00001 ± 0.00001b	0.00036 ± 0.00028c	0.00384 ± 0.00018c	0.00014 ± 0.00015b
	N200S100	0.10989 ± 0.02322c	0.0013 ± 0.00051b	0.00019 ± 0.00007b	0.00017 ± 0.00007a	0.0001 ± 0.00001a	0.00026 ± 0.00011a	0.00052 ± 0.00015b	0.00009 ± 0.00001a	0.01793 ± 0.00259b	0.00016 ± 0.0001a	0.00846 ± 0.00147b	0.02552 ± 0.01362b	0.00035 ± 0.00019a
	N200S200	0.01758 ± 0.00246c	0.00472 ± 0.00152a	0.00112 ± 0.00028a	0.00003 ± 0.00001b	0.00018 ± 0.00007a	0.00075 ± 0.0003a	0.00193 ± 0.00066a	0.00003 ± 0.00001b	0.08734 ± 0.03239b	0.00012 ± 0.00002a	0.00388 ± 0.00352b	0.24784 ± 0.01066a	0.00018 ± 0.00006a
	*P*(N)	*	*	*	ns	*	*	*	*	ns	*	*	*	*
	*P*(S)	*	*	*	ns	ns	ns	*	*	*	ns	ns	*	ns
	*P*(N*S)	ns	*	*	*	ns	ns	*	*	ns	ns	ns	*	*

Different letters indicate that the values differ significantly at *p* < 0.05. *indicates *p* < 0.05, ns indicates no significant difference.

**Table 7:** Effect of different fertilization treatments on the relative abundance of specific bacteria assemblages.

		*Acidibacter*	*Bacillus*	*Ensifer*	*Faecalibaculum*	*Gemmatimonas*	*Geobacter*	*Haliangium*	*Iamia*	*Lachnoclostridium*	*Lacibacter*	*Niastella*	*Ohtaekwangia*	*Rubrobacter*	*Streptomyces*	*unidentified_Nitrospiraceae*	*unidentified_Pryinomonadaceae*
Treatments	CK	0.00393 ± 0.00014a	0.00767 ± 0.00005b	0.0016 ± 0.00005b	0.00845 ± 0.00071a	0.00202 ± 0.00004a	0.00345 ± 0.00026ab	0.00543 ± 0.00048a	0.00072 ± 0b	0.00117 ± 0.00068b	0.0008 ± 0.00008b	0.00181 ± 0.00006b	0.00576 ± 0.00034b	0.00166 ± 0.00006b	0.00122 ± 0.00001b	0.00068 ± 0.00004c	0.00221 ± 0.00005a
	N100S100	0.00319 ± 0.0002b	0.00856 ± 0.00035a	0.00242 ± 0.00026a	0.00121 ± 0.00006bc	0.00124 ± 0.00022b	0.00524 ± 0.00105a	0.00358 ± 0.00039b	0.0012 ± 0.0002a	0.01757 ± 0.00216a	0.00173 ± 0.00061a	0.00326 ± 0.00077a	0.00808 ± 0.00068a	0.00295 ± 0.00029a	0.00199 ± 0.00038a	0.00045 ± 0.00008c	0.00142 ± 0.00018b
	N100S200	0.00264 ± 0.00021c	0.0097 ± 0.00136a	0.0017 ± 0.00013a	0.00339 ± 0.00464abc	0.00124 ± 0.00016b	0.00583 ± 0.00237a	0.00289 ± 0.0004b	0.00083 ± 0.00016a	0.01923 ± 0.01493a	0.00102 ± 0.00004a	0.00246 ± 0.00028b	0.00717 ± 0.00009a	0.00201 ± 0.00033a	0.00197 ± 0.00057a	0.00043 ± 0.00006c	0.0016 ± 0.00011a
	N200S100	0.00398 ± 0.00007a	0.00145 ± 0.00012c	0.00184 ± 0.00049b	0.0074 ± 0.00008ab	0.002 ± 0.00039a	0.00219 ± 0.00107b	0.0039 ± 0.00052a	0.00059 ± 0.00006b	0.0012 ± 0.00025b	0.00041 ± 0.00008b	0.00267 ± 0.00008a	0.00402 ± 0.00082c	0.00137 ± 0.00016ab	0.00068 ± 0.00009b	0.00118 ± 0.00016b	0.00138 ± 0.00026b
	N200S200	0.00415 ± 0.00028a	0.00124 ± 0.00008c	0.00113 ± 0.00007b	0.00031 ± 0.00012c	0.00255 ± 0.00016a	0.00082 ± 0.00014b	0.00582 ± 0.00037a	0.00069 ± 0.00013b	0.00137 ± 0.00023b	0.00027 ± 0.00007b	0.00192 ± 0.00026b	0.00258 ± 0.00075c	0.00177 ± 0.00006ab	0.00086 ± 0.0001b	0.00171 ± 0.00027a	0.00217 ± 0.00044a
	*P*(N)	*	*	*	ns	*	*	*	*	*	*	ns	*	*	*	*	ns
	*P*(S)	ns	ns	*	ns	ns	ns	ns	ns	ns	ns	*	*	ns	ns	*	*
	*P*(N*S)	*	ns	ns	*	ns	ns	*	*	ns	ns	ns	ns	*	ns	*	ns

Different letters indicate that the values differ significantly at *p* < 0.05. *indicates *p* < 0.05, ns indicates no significant difference.

### Relationships between microbial community composition and environmental factors

**Figure 7:**
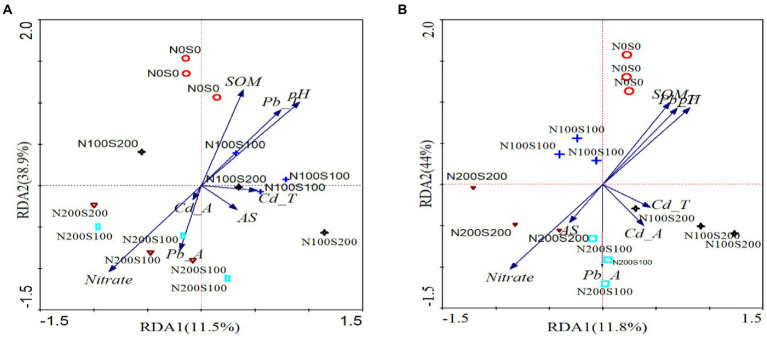
The RDA analysis of fungal **(A)**, Bacterrial **(B)** community and soil properties. pH (pH), soil organic matter (SOM), nitrate nitrogen (Nitrate), ammonium nitrogen (Animonium), soil total Pb (Pb-T), the content of acid-solube Pb (Pb-A), soil total Cd (Cd-T), and the content of acid-solube Cd (Cd-A).

**Figure 8:**
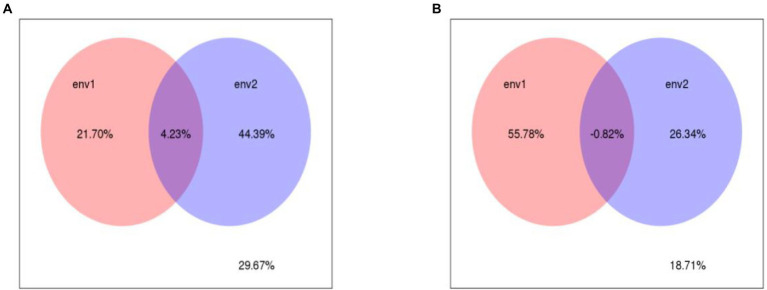
The VPA analysis of fungal **(A)**, Bacterrial **(B)** community and soil properties. env1 (The content of different speciation Pb) and env2 (The content of different speciation Cd).

RDA and VPA were used to analyze the comprehensive contribution of soil chemistry and heavy metals to bacteria and fungi (Figures 7, 8). The first and second RDA axes explained 11.5 and 38.9% of the fungal community variables, respectively. Based on RDA, the pH (*R*^2^ = 0.49), nitrate content (*R*^2^ = 0.48), and Cd content (*R*^2^ = 0.43) were the key factors affecting the fungal community in rhizosphere soil (*p* < 0.05; Figure 7A). VPA indicated that the Pb and Cd contents explained 21.7 and 44.3% of the total variation of the fungal community, respectively, and the interaction between Pb and Cd contents accounted for 4.23% of the total variation (Figure 8A). The first and second RDA axes explained 11.8 and 44% of the variance in the bacterial community, respectively. RDA indicated that pH (*R*^2^ = 0.61), nitrate content (*R*^2^ = 0.5), and Pb content (*R*^2^ = 0.38) were the key factors affecting the bacterial community in rhizosphere soil (*p* < 0.05; Figure 7B). VPA showed that the Pb and Cd contents, respectively, explained 55.78 and 26.34% of the total variation in the bacterial community (Figure 8B).

**Figure 9:**
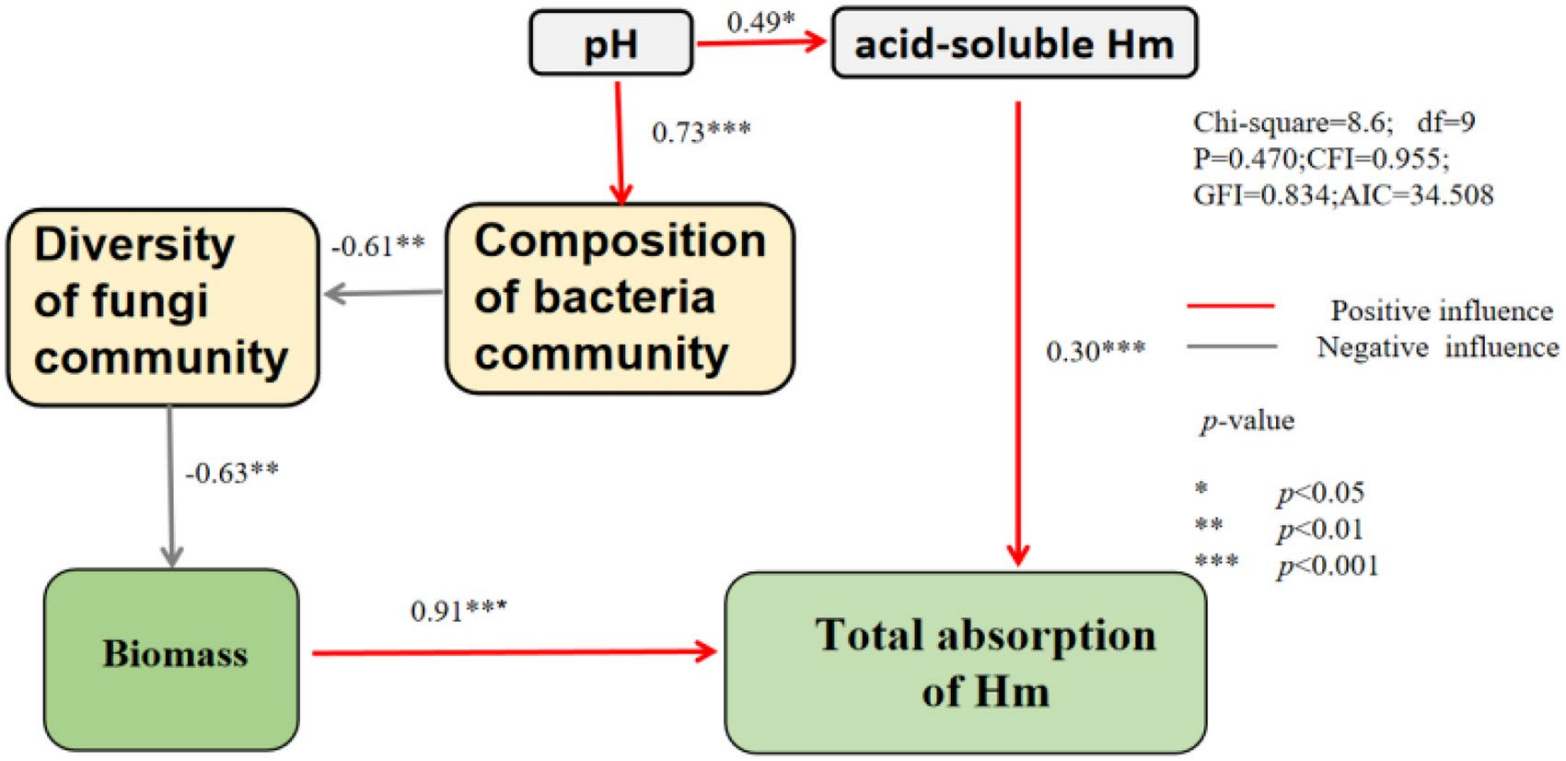
Structural equational model on the relationship between soil properties ˎmicrobial community and the total absorption of heavy metal.

The SEM model showed that pH had significant positive effects on the cotent of acid-soluble and composition of bacteria community (Figure 9). Bacterial communities alter fungal diversity and thereby affect plant biomass and heavy metal uptake. The content of acid-soluble heavy metals was positively correlated with the total absorption of heavy metals.

## Discussion

### Effects of N and S fertilizers on soil parameters and the total absorption of soil heavy metals

In this study, the decrease in pH was likely due to the application of N fertilizer, which stimulated nitrification and caused soil acidification (Zhou et al., 2015). Additionally, the contents of nitrate, AP and AS also increased after the application of N and S fertilizers, whereas the effects on soil chemical properties became more significant as the concentrations of N and S increased. These results might be attributed to changes in nutrients that improved microbial activity, stimulated nitrification, and increased the activity of soil phosphatase (Marklein et al., 2012; Tafazoli et al., 2019). Furthermore, the availability of N and S stimulate each other, the addition of S fertilizer reduced the leaching of soil nitrate, thereby improving the availability of N and increasing the content of AS in soil (Wang et al., 2016).

The phytoextraction of Pb and Cd by *S. integra* was enhanced under the N100S100 and N100S200 treatments, which can be attributed to the increased biomass and activity of heavy metals. Chai et al. (2018) reported that fertilizers enhanced soil fertility and prometed plant growth, which increased the accumulation of heavy metals. Fertilizers can also regulate microbial community to affect heavy metal absorption (Wang et al., 2020). The addition of N and S fertilizers can further increase the activity of heavy metal ions, and promote the accumulation of heavy metals in plants, however, excessive fertilizer can increase the activity of heavy metal ions to a level that causes toxic effects in plants themselves and inhibits plant growth (Wang et al., 2019).

### Bioavailability of soil heavy metals

In this study, the application of N and S fertilizers increased the bioavailability of soil heavy metal ions by reducing the soil pH, in agreement with previous studies thatis associated with enhanced heavy metal ion mobility and bioavailability (Gabarrón et al., 2018; Novak et al., 2018). Decreasing the SOM content also increased the solubility and activity of heavy metals, which might be due to a reduction in the number of metal adsorption sites and metal chelating agents (Venegas et al., 2015, 2016). Moreover, the application of N and S fertilizers increased the contents of acid-extracted Pb and Cd in rhizosphere soil but had no effect in non-rhizosphere soil, indicating that the heavy metal form was also affected by the root activity, particularly root exudates (Montiel-Rozas et al., 2016). Other studies have demonstrated that root exudates such as organic acids and amino acids can stimulate the conversion of insoluble heavy metal forms into soluble forms, thereby increasing the bioavailability and improving the remediation efficiency of plants (Luo et al., 2017; Liu et al., 2018b; Moshiri et al., 2019).

### Microbial community and metabolism

The application of N and S fertilizers reduced the diversity and richness of rhizosphere microorganisms. The structure of the rhizosphere microbial community varied significantly between different treatments, suggesting that soil microorganisms may adapt to different levels of nutrients in the environment by changing their community structure and composition (Zhou et al., 2017; Wang et al., 2018b; Li et al., 2019). The application of N and S fertilizers caused the Chao1 and Shannon indexes of the fungal community to decrease more than those of the bacterial community, consistent with Fierer et al. (2012), who found N application had little effect on bacterial community diversity. This may be because Pb and Cd are less toxic to the bacterial community than to the fungal community. Bacteria are more adaptable to abiotic stresses (e.g., heavy metals) than fungi, possibly due to the faster metabolism and higher substrate utilization of bacteria compared to fungi (David and Erland, 2016). Additionally, bacterial communities are dominant in soil and compete with fungi for nutrients (Deng et al., 2015). Changes in the microbial community structure can lead to changes in microbial metabolic functions (Tipayno et al., 2018). In this study, N and S fertilization increased the utilization rate of carbon sources. The utilization rate of CG in rhizosphere soil was greater than that in bulk soil, while the utilization rates of AG and CG in rhizosphere soil were lower than those in bulk soil. These results suggest that different microbial communities formed in the rhizosphere and bulk soils as a result of plant root activity. Compared with the bulk soil, the metabolic activity of rhizosphere microorganisms was significantly improved after fertilization, indicating that the establishment of beneficial interactions of microorganisms with plants, which was beneficial to improve phytoremediation efficiency. Microbial variation is closely related to the rhizosphere environment, leading to differences in microbial metabolism (Sasse et al., 2018). These changes have significant effects on the activation of heavy metals and the promotion of phytoremediation. In this study, RDA analysis indicated that the pH and nitrate content had significant effects on the structure of the rhizosphere microbial community. The composition of the microbial community changes in long-term contaminated soil, and the changes depend on the heavy metals and chemical properties of the soil (Zhang et al., 2016; Fajardo et al., 2018). Therefore, the application N and S fertilizers influences the absorption of heavy metals by affecting the metabolic activity and metabolic mode of microorganisms. The rhizosphere pH and the content of nitrate might play key roles in phytoremediation by *S. integra*.

### Changes in dominant and special microbes

The dominant bacterial species in this study were the same under different fertilization treatments; however, the relative abundances of different species were different. This may be due to the selection of microorganisms in response to changes in the rhizosphere environment. Increasing the bioavailability of heavy metals may affect the diversity of microorganisms by suppressing sensitive species that lack sufficient tolerance to heavy metal stress while also stimulating heavy metal-tolerant species (Xie et al., 2016). Under all treatments, the dominant fungal phyla were *Ascomycota* and *Basidiomycota*, with the relative abundance of *Ascomycota* exceeding 65%. These species play an important role in improving the tolerance of the host to heavy metals (Wang et al., 2021). In this study, N and S fertilizers increased the relative abundance of *Ascomycota* in rhizosphere soil. We speculate that the application of N and S fertilizers might affect mycorrhizal symbiosis, thereby influencing the resistance of *S. integra* to heavy metals. The relative abundance of *Proteobacteria* exceeded 29.5% under the different treatments. Guo et al. (2019) reported that *Proteobacteria* may be the most metal-resistant bacteria found in sites contaminated with heavy metals. Therefore, we speculate that *Proteobacteria* plays an important role in the restoration of heavy metal-contaminated soils by *S. integra*. However, the relative abundance of *Acidobacteria*, which was the second most abundant phylum, was suppressed by N and S fertilization, and its function cannot be ignored. Xu et al. (2021) suggested that *Acidobacteria* may resist or transform metal(loid)s in metal-contaminated sites. In this study, the relative abundance of *Bacteroidetes* was also increased by the application of N and S fertilizers, and Lin et al. (2019) reported that *Bacteroidetes* are the most dominant microbial group in heavy metal-contaminated soil. *Actinobacteria* are considered to be the key taxon in contaminated soil (Chao et al., 2016; Jiao et al., 2016; Hou et al., 2018; Samiran et al., 2018). These detected bacteria can reduce metal toxicity, prevent and control soil diseases, and adapt to extreme environments and environmental pressures (Li et al., 2015; Guo et al., 2019). Therefore, to improve the phytoremediation efficiency, rhizosphere microorganisms can be added to the rhizosphere of fast-growing woody plants.

At the genus level, some special fungi are pathogenic fungi such as *Fusarium* (Wen et al., 2020). They were less enriched in treatments (N100S100, N100S200), which promote phytoremediation. Thus, we speculate that the simultaneous application of N and S fertilizers improve the rhizosphere health of *S. integra* and promote its resistance to pathogens. Other special bacterial species that are enriched by fertilization may promote phytoremediation and play an important role in the remediation of contaminated soil. For example, *Streptomyces* produces siderophores to improve the absorption of certain nutrients by plants, and the secreted IAA can also stimulate plant growth (Huang et al., 2014), *Bacillus* and *Ensifer* have been reported to promote rhizosphere growth (Mesa et al., 2017; Sajid et al., 2020). Inoculating rhizosphere growth-promoting bacteria improve the repair efficiency of plants (Ke et al., 2021) and interact beneficially with AMF to enhance the extraction of heavy metals by plants (Mahohi and Raiesi, 2019). Some beneficial rhizosphere genera such as *Acidibacter* and *Streptomyces* significantly increased Cd uptake by *S. alfredii* (Yang et al., 2019). Other indigenous microbial communities also play important roles in the absorption of heavy metals by *S. integra*. (Niu et al., 2020). The mutually beneficial relationships between bacteria and fungi can be taken advantage of to improve the heavy metal tolerance of plants and assist in the phytoremediation of heavy metal-contaminated soil. We can infer that the application of N and S fertilizers affects the absorption of Pb and Cd by regulating the rhizosphere microbial composition.

## Conclusion

The application of N and S fertilizers affected the pH and nitrate content in the rhizosphere environment, increased the bioavailability of heavy metals, changed the utilization patterns of carbon sources by microbes, and altered the structure of the microbial community to affect the absorption of Pb and Cd by *S. integra*. Moreover, the application of N and S fertilizers can limit soil-born fungal diseases and enrich rhizosphere growth-promoting bacteria to improve the phytoremediation efficiency. Our findings provide a possible strategy for the plant-microbial remediation of polluted soil using fast-growing trees.

## Data availability statement

The datasets generated for this study can be found in https://www.ncbi.nlm.nih.gov/sra/PRJNA846123 and PRJNA846147, respectively.

## Author contributions

SW: methodology, visualization, investigation, formal analysis, and writing–original draft. XN: software, writing– review and editing, and supervision. DD: formal analysis and methodology. DH: resources, writing–review and editing, and supervision. All authors contributed to the article and approved the submitted version.

## Funding

This research was funded by Natural Science Foundation of Hebei province (No. C2022204161), Basic Scientific Research Funds of Hebei Provincial colleges and universities (No. KY2021053), Youth Fund Project of Hebei Provincial Education Department (No. QN2022120), and Subjects Group of Modern Forestry (No. XK1008601519). This research was also partly supported by the National Natural Science Foundation of China (Nos. 31971651 and 31600486).

## Conflict of interest

The authors declare that the research was conducted in the absence of any commercial or financial relationships that could be construed as a potential conflict of interest.

## Publisher’s note

All claims expressed in this article are solely those of the authors and do not necessarily represent those of their affiliated organizations, or those of the publisher, the editors and the reviewers. Any product that may be evaluated in this article, or claim that may be made by its manufacturer, is not guaranteed or endorsed by the publisher.

## Supplementary material

The Supplementary Material for this article can be found online at: https://www.frontiersin.org/articles/10.3389/fmicb.2022.945847/full#supplementary-material
