# Supplementary material for: Nitrogen and sulfur fertilizers promote the absorption of lead and cadmium with Salix integra Thunb. by increasing the bioavailability of heavy metals and regulating rhizosphere microbes
Source: Front Microbiol. 2022 Aug 3;13:945847. doi: 10.3389/fmicb.2022.945847 (PMC9383694; doi:10.3389/fmicb.2022.945847)
Supplement: Supplementary file 1 [file Data_Sheet_2.docx]

| **Tab S1.** fertilization treatments | | | |
| --- | --- | --- | --- |
| Treatments | Nitrogen (kg·ha^-1^·year^-1^） | | Sulfur (kg·ha^-1^·year^-1^） |
| N0S0 | 0 | 0 | |
| N0S100 | 0 | 100 | |
| N0S200 | 0 | 200 | |
| N100S0 | 100 | 0 | |
| N100S100 | 100 | 100 | |
| N100S200 | 100 | 200 | |
| N200S0 | 200 | 0 | |
| N200S100 | 200 | 100 | |
| N200S200 | 200 | 200 | |

| **Tab S2.** Topological properties of the networks |
| --- |
| \| Id \| Label \| degree \| weighted degree \| Eccentricity \| closnesscentrality \| harmonicclosnesscentrality \| betweenesscentrality \| Authority \| Hub \| modularity_class \| \| --- \| --- \| --- \| --- \| --- \| --- \| --- \| --- \| --- \| --- \| --- \| \| 1 \| OTU5 \| 1 \| 0.961538 \| 1 \| 1 \| 1 \| 0 \| 0 \| 0 \| 8 \| \| 2 \| OTU8 \| 1 \| 0.983517 \| 1 \| 1 \| 1 \| 0 \| 0 \| 0 \| 1 \| \| 3 \| OTU27 \| 1 \| 0.953168 \| 1 \| 1 \| 1 \| 0 \| 0 \| 0 \| 0 \| \| 4 \| OTU28 \| 1 \| 0.983517 \| 1 \| 1 \| 1 \| 0 \| 0 \| 0 \| 1 \| \| 5 \| OTU29 \| 1 \| 0.960111 \| 2 \| 0.666667 \| 0.75 \| 0 \| 0 \| 0 \| 2 \| \| 6 \| OTU39 \| 1 \| 0.93113 \| 1 \| 1 \| 1 \| 0 \| 0 \| 0 \| 3 \| \| 7 \| OTU41 \| 1 \| 0.935352 \| 1 \| 1 \| 1 \| 0 \| 0 \| 0 \| 4 \| \| 8 \| OTU45 \| 2 \| 1.885826 \| 2 \| 0.6 \| 0.666667 \| 5 \| 0.242723 \| 0.242723 \| 13 \| \| 9 \| OTU50 \| 1 \| 0.948974 \| 1 \| 1 \| 1 \| 0 \| 0 \| 0 \| 5 \| \| 10 \| OTU55 \| 2 \| 1.868132 \| 1 \| 1 \| 1 \| 1 \| 0 \| 0 \| 6 \| \| 11 \| OTU59 \| 2 \| 1.85806 \| 3 \| 0.545455 \| 0.638889 \| 0 \| 0.382979 \| 0.382979 \| 13 \| \| 12 \| OTU63 \| 1 \| 0.92985 \| 1 \| 1 \| 1 \| 0 \| 0 \| 0 \| 7 \| \| 13 \| OTU68 \| 1 \| 0.937588 \| 3 \| 0.4 \| 0.472222 \| 0 \| 0.085705 \| 0.085705 \| 13 \| \| 14 \| OTU88 \| 3 \| 2.781537 \| 3 \| 0.6 \| 0.722222 \| 0.5 \| 0.482919 \| 0.482919 \| 13 \| \| 15 \| OTU89 \| 1 \| 0.961538 \| 1 \| 1 \| 1 \| 0 \| 0 \| 0 \| 8 \| \| 16 \| OTU98 \| 1 \| 0.937767 \| 1 \| 1 \| 1 \| 0 \| 0 \| 0 \| 9 \| \| 17 \| OTU103 \| 1 \| 0.937767 \| 1 \| 1 \| 1 \| 0 \| 0 \| 0 \| 9 \| \| 18 \| OTU110 \| 1 \| 0.928375 \| 1 \| 1 \| 1 \| 0 \| 0 \| 0 \| 10 \| \| 19 \| OTU121 \| 1 \| 0.935352 \| 1 \| 1 \| 1 \| 0 \| 0 \| 0 \| 4 \| \| 20 \| OTU125 \| 1 \| 0.926717 \| 1 \| 1 \| 1 \| 0 \| 0 \| 0 \| 25 \| \| 21 \| OTU129 \| 2 \| 1.906177 \| 1 \| 1 \| 1 \| 1 \| 0 \| 0 \| 2 \| \| 22 \| OTU130 \| 2 \| 1.860629 \| 3 \| 0.545455 \| 0.638889 \| 0 \| 0.382979 \| 0.382979 \| 13 \| \| 23 \| OTU136 \| 1 \| 0.931133 \| 1 \| 1 \| 1 \| 0 \| 0 \| 0 \| 15 \| \| 24 \| OTU147 \| 1 \| 0.946066 \| 2 \| 0.666667 \| 0.75 \| 0 \| 0 \| 0 \| 2 \| \| 25 \| OTU162 \| 1 \| 0.944613 \| 1 \| 1 \| 1 \| 0 \| 0 \| 0 \| 23 \| \| 26 \| OTU188 \| 1 \| 0.928375 \| 1 \| 1 \| 1 \| 0 \| 0 \| 0 \| 10 \| \| 27 \| OTU225 \| 1 \| 0.953172 \| 1 \| 1 \| 1 \| 0 \| 0 \| 0 \| 11 \| \| 28 \| OTU235 \| 1 \| 0.934066 \| 2 \| 0.666667 \| 0.75 \| 0 \| 0 \| 0 \| 6 \| \| 29 \| OTU243 \| 1 \| 0.947587 \| 1 \| 1 \| 1 \| 0 \| 0 \| 0 \| 21 \| \| 30 \| OTU252 \| 1 \| 0.953172 \| 1 \| 1 \| 1 \| 0 \| 0 \| 0 \| 11 \| \| 31 \| OTU264 \| 1 \| 0.992857 \| 1 \| 1 \| 1 \| 0 \| 0 \| 0 \| 26 \| \| 32 \| OTU275 \| 1 \| 0.935173 \| 1 \| 1 \| 1 \| 0 \| 0 \| 0 \| 12 \| \| 33 \| OTU295 \| 1 \| 0.92985 \| 1 \| 1 \| 1 \| 0 \| 0 \| 0 \| 7 \| \| 34 \| OTU305 \| 1 \| 0.929273 \| 1 \| 1 \| 1 \| 0 \| 0 \| 0 \| 14 \| \| 35 \| OTU306 \| 1 \| 0.935173 \| 1 \| 1 \| 1 \| 0 \| 0 \| 0 \| 12 \| \| 36 \| OTU309 \| 5 \| 4.681365 \| 2 \| 0.857143 \| 0.916667 \| 11.5 \| 0.601702 \| 0.601702 \| 13 \| \| 37 \| OTU312 \| 1 \| 0.934449 \| 1 \| 1 \| 1 \| 0 \| 0 \| 0 \| 22 \| \| 38 \| OTU323 \| 1 \| 0.929273 \| 1 \| 1 \| 1 \| 0 \| 0 \| 0 \| 14 \| \| 39 \| OTU342 \| 1 \| 0.933888 \| 1 \| 1 \| 1 \| 0 \| 0 \| 0 \| 17 \| \| 40 \| OTU407 \| 1 \| 0.948974 \| 1 \| 1 \| 1 \| 0 \| 0 \| 0 \| 5 \| \| 41 \| OTU449 \| 1 \| 0.930362 \| 1 \| 1 \| 1 \| 0 \| 0 \| 0 \| 27 \| \| 42 \| OTU457 \| 2 \| 1.884459 \| 1 \| 1 \| 1 \| 1 \| 0 \| 0 \| 18 \| \| 43 \| OTU459 \| 1 \| 0.931133 \| 1 \| 1 \| 1 \| 0 \| 0 \| 0 \| 15 \| \| 44 \| OTU476 \| 1 \| 0.941045 \| 3 \| 0.5 \| 0.555556 \| 0 \| 0.21246 \| 0.21246 \| 13 \| \| 45 \| OTU511 \| 1 \| 0.940608 \| 1 \| 1 \| 1 \| 0 \| 0 \| 0 \| 16 \| \| 46 \| OTU574 \| 1 \| 0.953168 \| 1 \| 1 \| 1 \| 0 \| 0 \| 0 \| 0 \| \| 47 \| OTU585 \| 1 \| 0.943143 \| 1 \| 1 \| 1 \| 0 \| 0 \| 0 \| 20 \| \| 48 \| OTU603 \| 1 \| 0.941861 \| 1 \| 1 \| 1 \| 0 \| 0 \| 0 \| 19 \| \| 49 \| OTU755 \| 1 \| 0.940608 \| 1 \| 1 \| 1 \| 0 \| 0 \| 0 \| 16 \| \| 50 \| OTU787 \| 1 \| 0.933888 \| 1 \| 1 \| 1 \| 0 \| 0 \| 0 \| 17 \| \| 51 \| OTU1076 \| 1 \| 0.932235 \| 1 \| 1 \| 1 \| 0 \| 0 \| 0 \| 29 \| \| 52 \| OTU1222 \| 1 \| 0.954609 \| 2 \| 0.666667 \| 0.75 \| 0 \| 0 \| 0 \| 18 \| \| 53 \| OTU1878 \| 1 \| 0.941861 \| 1 \| 1 \| 1 \| 0 \| 0 \| 0 \| 19 \| \| 54 \| OTU3594 \| 1 \| 0.943143 \| 1 \| 1 \| 1 \| 0 \| 0 \| 0 \| 20 \| \| 55 \| OTU4333 \| 1 \| 0.93113 \| 1 \| 1 \| 1 \| 0 \| 0 \| 0 \| 3 \| \| 56 \| OTU5746 \| 1 \| 0.947587 \| 1 \| 1 \| 1 \| 0 \| 0 \| 0 \| 21 \| \| 57 \| OTU5864 \| 1 \| 0.934449 \| 1 \| 1 \| 1 \| 0 \| 0 \| 0 \| 22 \| \| 58 \| OTU6303 \| 1 \| 0.944613 \| 1 \| 1 \| 1 \| 0 \| 0 \| 0 \| 23 \| \| 59 \| OTU6523 \| 1 \| 0.943314 \| 1 \| 1 \| 1 \| 0 \| 0 \| 0 \| 24 \| \| 60 \| OTU6524 \| 1 \| 0.934066 \| 2 \| 0.666667 \| 0.75 \| 0 \| 0 \| 0 \| 6 \| \| 61 \| OTU6769 \| 1 \| 0.92985 \| 2 \| 0.666667 \| 0.75 \| 0 \| 0 \| 0 \| 18 \| \| 62 \| OTU7766 \| 1 \| 0.943314 \| 1 \| 1 \| 1 \| 0 \| 0 \| 0 \| 24 \| \| 63 \| OTU7883 \| 1 \| 0.926717 \| 1 \| 1 \| 1 \| 0 \| 0 \| 0 \| 25 \| \| 64 \| OTU7913 \| 1 \| 0.969701 \| 1 \| 1 \| 1 \| 0 \| 0 \| 0 \| 33 \| \| 65 \| OTU7916 \| 1 \| 0.927099 \| 4 \| 0.4 \| 0.520833 \| 0 \| 0 \| 0 \| 28 \| \| 66 \| OTU7921 \| 1 \| 0.928191 \| 4 \| 0.4 \| 0.520833 \| 0 \| 0 \| 0 \| 28 \| \| 67 \| OTU7924 \| 1 \| 0.992857 \| 1 \| 1 \| 1 \| 0 \| 0 \| 0 \| 26 \| \| 68 \| OTU7940 \| 1 \| 0.930362 \| 1 \| 1 \| 1 \| 0 \| 0 \| 0 \| 27 \| \| 69 \| OTU7950 \| 2 \| 1.860983 \| 3 \| 0.571429 \| 0.708333 \| 3 \| 0 \| 0 \| 28 \| \| 70 \| OTU7951 \| 2 \| 1.910802 \| 1 \| 1 \| 1 \| 0 \| 0 \| 0 \| 30 \| \| 71 \| OTU7980 \| 1 \| 0.932235 \| 1 \| 1 \| 1 \| 0 \| 0 \| 0 \| 29 \| \| 72 \| OTU7982 \| 2 \| 1.874418 \| 2 \| 0.666667 \| 0.75 \| 4 \| 0 \| 0 \| 28 \| \| 73 \| OTU7990 \| 1 \| 0.948966 \| 1 \| 1 \| 1 \| 0 \| 0 \| 0 \| 32 \| \| 74 \| OTU8020 \| 2 \| 1.926436 \| 1 \| 1 \| 1 \| 0 \| 0 \| 0 \| 30 \| \| 75 \| OTU8117 \| 2 \| 1.913313 \| 1 \| 1 \| 1 \| 0 \| 0 \| 0 \| 30 \| \| 76 \| OTU8154 \| 1 \| 0.998213 \| 1 \| 1 \| 1 \| 0 \| 0 \| 0 \| 31 \| \| 77 \| OTU8380 \| 1 \| 0.998213 \| 1 \| 1 \| 1 \| 0 \| 0 \| 0 \| 31 \| \| 78 \| OTU8964 \| 2 \| 1.868725 \| 3 \| 0.571429 \| 0.708333 \| 3 \| 0 \| 0 \| 28 \| \| 79 \| OTU9627 \| 1 \| 0.948966 \| 1 \| 1 \| 1 \| 0 \| 0 \| 0 \| 32 \| \| 80 \| OTU10025 \| 1 \| 0.969701 \| 1 \| 1 \| 1 \| 0 \| 0 \| 0 \| 33 \| |

| Table S3.Concentrations (mean) of heavy metals in root,stem,leaves |
| --- |
| \| Treatments \| Pb \| \| \|  \| Cd \|  \| \| --- \| --- \| --- \| --- \| --- \| --- \| --- \| \| Root/ mg·kg^-1^ \| Stem/ mg·kg^-1^ \| Leaf/ mg·kg^-1^ \| Root/ mg·kg^-1^ \| Stem/ mg·kg^-1^ \| Leaf/ mg·kg^-1^ \| \| N0S0 \| (26.48±2.45)f \| (10.15±0.99)bc \| (11.94±0.56)a \| (35.42±2.59)e \| (33.04±3.05)abc \| (66.05±0.56)a \| \| N0S100 \| (22.29±2.52)g \| (8.38±0.67)cd \| (8.86±1.27)bc \| (42.88±4.04)cd \| (34.08±3.26)abc \| (51.36±1.27)b \| \| N0S200 \| (24.65±1.01)g \| (8.27±0.70)cd \| (11.28±0.82)a \| (38.55±4.88)de \| (28.41±2.08)c \| (36.33±0.82)cd \| \| N100S0 \| (30.56±0.09)e \| (9.29±1.20)cd \| (11.5±0.31)a \| (49.21±1.66)bc \| (31.73±2.55)bc \| (45.93±0.31)bc \| \| N100S100 \| (30.01±1.42)e \| (7.46±0.79)d \| (10.36±0.11)ab \| (57.03±5.87)a \| (27.09±1.08)c \| (47.85±0.11)bc \| \| N100S200 \| (51.78±2.03)b \| (8.91±1.24)cd \| (8.72±0.76)bc \| (44.06±1.39)cd \| (37.8±0.52)ab \| (66.38±0.76)a \| \| N200S0 \| (34.29±0.65)d \| (9.80±0.90)c \| (9.04±1.14)bc \| (42.26±0.42)cde \| (39.55±1.51)a \| (66.03±1.14)a \| \| N200S100 \| (55.28±0.95)a \| (11.76±0.25)a \| (5.95±0.39)d \| (50.95±0.45)ab \| (28.42±0.64)c \| (43.27±0.39)bcd \| \| N200S200 \| (47.32±1.07)c \| (14.01±0.30)a \| (7.81±0.16)e \| (37.76±0.26)de \| (27.88±0.69)c \| (33.01±0.16)d \| |
